# Assessment of Clinical Effectiveness of BNT162b2 COVID-19 Vaccine in US Adolescents

**DOI:** 10.1001/jamanetworkopen.2022.0935

**Published:** 2022-03-03

**Authors:** Carlos R. Oliveira, Linda M. Niccolai, Hassan Sheikha, Lina Elmansy, Chaney C. Kalinich, Nathan D. Grubaugh, Eugene D. Shapiro

**Affiliations:** 1 Department of Pediatrics, Section of Infectious Diseases and Global Health, Yale School of Medicine, New Haven, Connecticut; 2 Department of Biostatistics, Section of Health Informatics, Yale School of Public Health, New Haven, Connecticut; 3 Department of Epidemiology of Microbial Diseases, Yale School of Public Health, New Haven, Connecticut; 4 Department of Ecology and Evolutionary Biology, Yale University, New Haven, Connecticut

## Abstract

**Importance:**

The emergence of the B.1.617.2 (Delta) variant of SARS-CoV-2 has led to increases in both infections and hospitalizations among adolescents. Little is known about the effectiveness of the BNT162b2 vaccine in adolescents in the general population, as opposed to a clinical trial population.

**Objective:**

To estimate the effectiveness of the BNT162b2 vaccine in adolescents aged 12 to 18 years.

**Design, Setting, and Participants:**

This was a matched case-control study among adolescents (aged 12-18 years) who had results from a SARS-CoV-2 reverse transcription–polymerase chain reaction (RT-PCR) test. Immunization histories, relevant clinical data, and RT-PCR test results were obtained from the Yale New Haven Health System’s medical records between June 1, 2021, and August 15, 2021, when the Delta variant caused 92% of infections in Connecticut. Case participants were defined as adolescents who had a positive test result and an associated medical encounter. Control participants were defined as those who had a negative test result and were matched to a case participant by age, county of residence, and date of testing.

**Exposures:**

Adolescents were defined as fully immunized if they had received 2 doses of vaccine at least 14 days before focal time.

**Main Outcomes and Measures:**

The primary outcome measured was SARS-CoV-2 infection confirmed by RT-PCR. The vaccine’s effectiveness (VE) was estimated using matched odds ratios from conditional logistic regression models. Secondary measures included estimated VE by clinical symptoms, number of vaccine doses received, and elapsed time from immunization.

**Results:**

A total of 6901 adolescents were tested for SARS-CoV-2. The final sample comprised 186 case participants and 356 matched control participants. The median age was 14 (IQR, 13-16) years, 262 (48%) identified as female, 81 (15%) as Black, 82 (15%) as Hispanic, and 297 (55%) as White. Overall, 134 (25%) were fully immunized (case participants, 10 [5%]; control participants, 124 [35%]). The median time between immunization and the SARS-CoV-2 test was 62 days (range, 17-129 days). Within 4 months of receiving 2 doses, VE against any infection was estimated to be 91% (95% CI, 80%-96%); against asymptomatic infection, 85% (95% CI, 57%-95%). Effectiveness after a single dose was estimated to be 74% (95% CI, 18%-92%).

**Conclusions and Relevance:**

In this retrospective case-control study of US adolescents, 2 doses of BNT162b2 vaccine appeared to provide excellent protection for at least 4 months after immunization against both symptomatic and asymptomatic SARS-CoV-2 infections.

## Key Points

**Question:**

What is the association between the BNT162b2 COVID-19 vaccine and SARS-CoV-2 positivity among adolescents?

**Findings:**

This case-control study of 542 adolescents was conducted when the Delta variant of SARS-CoV-2 was predominant and within 4 months of the vaccine rollout for adolescents. Overall, the estimated effectiveness of the BNT162b2 vaccine was 91%, with 93% protection against symptomatic infections and 85% effectiveness against asymptomatic infection.

**Meaning:**

These findings suggest that the BNT162b2 vaccine was effective in adolescents within 4 months of immunization, including against infections caused by the Delta variant.

## Teaser

This case-control study estimates the effectiveness of the BNT162b2 vaccine in adolescents aged 12 to 18 years.

## Introduction

Recent estimates from the American Academy of Pediatrics show that close to 6 million children younger than 18 years have been infected with SARS-CoV-2 in the United States alone, of whom 542 have died.^1^ The death toll of SARS-CoV-2 in children has surpassed that recorded from influenza during any season.^2^

As the COVID-19 pandemic has progressed, genetically distinct variants of SARS-CoV-2 have evolved. The more infectious B.1.617.2 lineage, also known as Delta, was the most common variant in the United States in the summer and fall of 2021.^3,4,5^ The increasing prevalence of the Delta variant has been accompanied by a sharply rising rate of hospitalization for COVID-19 among children younger than 18 years. Recent data from the US Centers for Disease Control and Prevention (CDC) showed that the hospitalization rate among children and adolescents increased 5-fold during the 6 weeks after the Delta variant became predominant (June 20 to July 31, 2021).^6^ Among those hospitalized, approximately a quarter were admitted to an intensive care unit, and 1.8% died.

On December 11, 2020, the US Food and Drug Administration (FDA) issued an Emergency Use Authorization (EUA) for the BNT162b2 COVID-19 vaccine (Pfizer-BioNTech) for individuals aged 16 years or older,^7^ which was expanded to include children aged 12 to 15 years or older on May 10.^8^ This was based on a randomized, double-blind, placebo-controlled clinical trial of 2200 adolescents that found the vaccine’s efficacy was 100% (95% CI, 75%-100%) against symptomatic, laboratory-confirmed COVID-19 in this age group.^9^

The degree of protection a vaccine provides in in the general population, as opposed to a clinical trial population, (ie, its effectiveness) does not always equate to its efficacy in the controlled setting of a clinical trial.^10^ It is difficult to replicate the rigorous protocols of a vaccine trial in normal clinical practice. Various factors, including underlying health conditions, storage of vaccines (eg, ultracold storage), adherence to the dosing schedules, and changes in the circulating strains, could significantly influence the vaccine’s protective effect in real-world settings.

Following the introduction of the BNT162b2 COVID-19 vaccine, several studies have confirmed the vaccine’s effectiveness in the adult population and among older adults.^11,12,13,14,15,16,17,18^ However, there are relatively few data on the vaccine’s protective effect in children 18 years or younger in the United States. As of July 31, 2021, less than one-third of adolescents in the United States had completed the COVID-19 vaccine series.^19^ One of the factors that parents and adolescents most commonly reported would increase their willingness to get vaccinated is having more information about the vaccine’s protective effect in children.^20^

To better characterize the benefits associated with the BNT162b2 COVID-19 vaccine in adolescents, we conducted a matched case-control study in a diverse US population with the primary aim of estimating the vaccine’s effectiveness (VE) at preventing SARS-CoV-2 infections. Secondary aims were to estimate the VE by the number of doses received, by time from immunization, and against asymptomatic infection.

## Methods

### Study Population

Participants were Connecticut residents aged 12 through 18 years who had a reverse transcriptase–polymerase chain reaction (RT-PCR) assay of a nasopharyngeal swab for SARS-CoV-2 between June 1 and August 15, 2021, and had an associated medical encounter in the Yale–New Haven Health System (YNHHS), where symptoms (or their absence) at the time of testing were noted. The YNHHS is one of the largest health care systems in the country, with close to 4 million outpatient encounters every year.^21^ This system is comprised of 5 large hospitals throughout Connecticut, including the Yale–New Haven Children’s Hospital, the largest full-service children’s hospital in the state, with more 35 000 pediatric emergency department encounters every year. The sites encompass a geographic area of approximately 650 square miles and serve a diverse patient population that closely resembles the nation in terms of race, ethnicity, and socioeconomic characteristics.^22^ All clinics, emergency departments, and inpatient units in the YNHHS use a single electronic health record (EHR) system.

The institutional review board at the Yale School of Medicine approved this study and waived the requirement for informed consent, as this research involved minimal risk to participants and used data collected for public health activities or routine clinical practice. This study followed the Strengthening the Reporting of Observational Studies in Epidemiology (STROBE) reporting guideline.

### Study Design

In this matched case-control study, case participants (those with a positive SARS-CoV-2 test result) and matched control participants (those with a negative SARS-CoV-2 test result) were identified using Impact of COVID-19 in Pediatrics at Yale (iCoPe-Yale), an ongoing registry that contains relevant clinical information from every child and adolescent aged 18 years or younger who underwent testing for SARS-CoV-2 and had an encounter (in person, by video, or by telephone) at a YNHHS site. The registry contains data on patients who were tested because of COVID-19–like symptoms as well as asymptomatic patients tested for other reasons, such as screening before a medical procedure, because of pending hospitalization or travel, either known or possible exposure to someone with COVID-19 (eg, contact tracing or return-to-school protocols), or for individual requests. The registry includes data on demographic characteristics, diagnoses, laboratory results, prescriptions ordered, and county of residence. Race and ethnicity were ascertained from the most recently stored variables for race and ethnicity in the EHR. Race and ethnicity were categorized into Hispanic, non-Hispanic Black, non-Hispanic White, and non-Hispanic other, which included American Indian or Alaska Native, Asian, Native Hawaiian, and Pacific Islander, and were collapsed in the analyses because of small numbers. Race and ethnicity were included in the analysis because they are potential confounders or effect modifiers of VE. Patients who explicitly opted out of research were not included in the registry. For this study, patients were excluded if they either were immunosuppressed (based on either recent diagnoses or medications they were receiving) or had documented SARS-CoV-2 infection before the study period (eTable 1 in Supplement 1).

Individuals with a positive result for SARS-CoV-2 infection by RT-PCR assay of a nasopharyngeal swab were included as case participants. As part of the Yale SARS-CoV-2 Genomic Surveillance Initiative, residual clinical specimens from patients with confirmed SARS-CoV-2 infections were obtained for genomic sequencing.^23^ Samples with a cycle threshold less than 35 were sequenced using the Illumina COVIDSeq protocol.^24^ Lineages were assigned using the most recent pangolin software.^25^ Additional details on genomic sequencing methods can be found in the eMethods in Supplement 1.

Patients who underwent testing for SARS-CoV-2 but whose test result was negative were included as potential control participants. A list of all potential control participants was created that individually matched each case by age (within 1 year), county of residence, and focal time (within 1 week). Focal time was defined either as the date of onset of symptoms for symptomatic cases or as the date of the SARS-CoV-2 test for asymptomatic cases. Symptomatic patients were those who had at least 1 COVID-19–like symptom at the time of testing (eTable 1 in Supplement 1). A random number generator was used to determine which 2 individually matched control participants were selected when more than 2 potential matched control participants were identified for a case participant. Calculations of sample size and statistical power are shown in the eMethods in Supplement 1.

### Vaccination Status

Investigators trained in medical record abstraction used a standardized form to collect immunization histories and relevant clinical data from electronic health records, using identical methods and efforts for both case and matched control participants. In Connecticut, all doses of SARS-CoV-2 vaccines administered are recorded within 24 hours of administration via that state’s immunization information system,^26^ which is automatically synced with the EHR of the YNHHS. Only doses of vaccines documented in the EHR were counted in these analyses. Case and matched control participants were defined as fully immunized if they had received 2 doses of vaccine at least 14 days before focal time. We defined individuals as partly immunized if they had received 1 dose of SARS-CoV-2 vaccine at least 14 days before focal time and either had not received a second dose or it was given less than 14 days before focal time. None of the adolescents in our study had received the mRNA-1273 (Moderna) or the Janssen (Johnson & Johnson) SARS-Cov-2 vaccines, so our analyses assessed the effectiveness only of BNT162b2.

### Statistical Analysis

To assess for balance in characteristics (covariates) between case and control participants, we used standardized mean differences (SMDs), calculated as the difference in means of a covariate between case and control participants divided by its pooled SD.^27^ The VE was estimated as VE = (1 − odds ratio) × 100%, using matched odds ratios (ORs) from a conditional logistic regression model in which vaccination status was the exposure and case or control status was the outcome, as previously described.^28^ The VE was first estimated for all patients irrespective of their symptoms or the reason for testing. To assess the VE against symptomatic and against asymptomatic infection, case participants were stratified based on whether they had at least 1 COVID-19–like symptom at the time of testing. Adjusted estimates of VE (aVE) were calculated using multivariable models that included covariates with a SMD greater than 0.2 on bivariate analysis, which were race and ethnicity, health insurance status, and history of exposure to SARS-CoV-2.

To explore possible heterogeneity in VE for the Delta variant, the unadjusted model was narrowed to include only case participants from whom the viruses were sequenced and identified as the Delta variant. To address uncertainty in the selection of the model, we tested several approaches to selecting variables (2-stage, backward selection, and stepwise change-in-estimate methods) and assessed results for consistency in the aVE estimates. To assess for the potential of residual bias, we compared the proportions of case participants and of control participants who had received an influenza vaccine during the previous respiratory virus season and before focal time, as has been previously described.^29,30,31,32^ Additional details regarding the logic behind our modeling approach and sensitivity analyses are provided in the eMethods in Supplement 1. Statistical analyses were conducted with the use of Stata/MP software version 17.0 (StataCorp).

## Results

### Study Population

**Figure 1.**
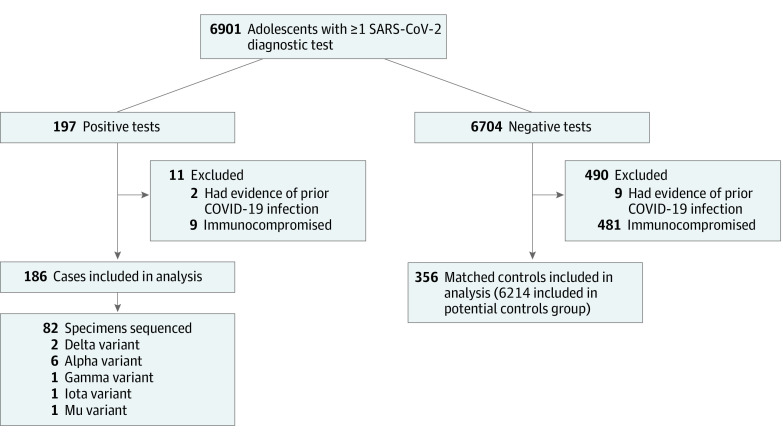
Flow Diagram for the Selection of Adolescents Shown is the flow for inclusion of adolescents in the matched case-control study.

A total of 6901 adolescents were tested for SARS-CoV-2 in the YNHHS between June 1, 2021, and August 19, 2021. Among the 197 adolescents who tested positive for SARS-CoV-2, 186 (94%) met inclusion criteria. Two closely matched control participants were identified for 170 case participants (91%); the remaining 16 case participants were each matched with 1 control participant (Figure 1). During the study period, 1455 of 1580 SARS-CoV-2 viral isolates (92%) sequenced from the YNHHS were identified as the Delta variant (eFigure 1 in Supplement 1). Of the 82 adolescent case participants from this study whose isolates that were sequenced, 73 (89%) were identified as the Delta variant. Most cases resided in either Fairfield (62 [33%]) or New Haven (92 [49%]) County (eFigure 2 in Supplement 1).

The characteristics of the adolescents are shown in Table 1 and Table 2. There were important differences in the proportions of case participants and of control participants who were asymptomatic (72 [39%] vs 323 [91%]), uninsured (31 [17%] vs 34 [10%]), of Hispanic ethnicity (37 [20%] vs 45 [13%]), or had a known exposure to someone with SARS-CoV-2 (136 [73%] vs 292 [82%]). There were no important differences in the distributions of age, sex, the median number of medical visits since January 1, 2020, or the presence of at least 1 comorbid illness (Table 1). Only 147 adolescents (27%) were tested for SARS-CoV-2 because of COVID-19–like symptoms. The remaining adolescents were screened because of an exposure to someone with SARS-CoV-2, preprocedural or travel screening, or individual requests (Table 2).

**Table 1.** Characteristics of the Adolescents

Characteristic	Participants, No. (%)			Standardized mean difference^a^
	Total (N = 542)	Case (n = 186)	Control (n = 356)	
Age, median (IQR), y	14 (13-16)	14 (13-16)	14 (13-16)	−0.09
Sex				
Female	262 (48.3)	86 (46.2)	176 (49.4)	−0.06
Male	280 (51.7)	100 (53.8)	180 (50.6)	0.06
Race or ethnic group				
Black, non-Hispanic	81 (14.9)	34 (18.3)	47 (13.2)	0.14
Hispanic or Latinx	82 (15.1)	37 (19.9)	45 (12.6)	0.20
White, non-Hispanic	297 (54.8)	92 (49.5)	205 (57.6)	−0.16
Other^b^	66 (12.2)	14 (7.5)	52 (14.6)	−0.23
Unknown	16 (3.0)	9 (4.8)	7 (2.0)	0.16
Health insurance				
Private	311 (57.4)	88 (47.3)	223 (62.6)	−0.31
Government	166 (30.6)	67 (36.0)	99 (27.8)	0.18
Uninsured or unknown	65 (12.0)	31 (16.7)	34 (9.6)	0.21
Medical history				
Any comorbidities^c^	190 (35.1)	63 (33.9)	127 (35.7)	−0.04
BMI >95 percentile	80 (14.8)	30 (16.1)	50 (14.0)	0.06
Month sample was obtained				
June	68 (12.5)	24 (12.9)	44 (12.4)	0.02
July	192 (35.4)	64 (34.4)	128 (36.0)	−0.03
August	282 (52.0)	98 (52.7)	184 (51.7)	0.02
BNT162b2 vaccination status				
Unvaccinated	388 (71.6)	173 (93.0)	215 (60.4)	0.83
1 Dose	20 (3.7)	3 (1.6)	17 (4.8)	−0.18
2 Doses	134 (24.7)	10 (5.4)	124 (34.8)	−0.79
Health care utilization				
≥1 Influenza vaccine dose^d^	132 (24.4)	45 (24.2)	87 (24.4)	−0.03
Medical visits after Jan 1, 2020, median (range)	3.0 (1.0 to 5.0)	2.0 (0.0 to 4.0)	3.0 (1.0 to 6.0)	−0.14

Abbreviation: BMI, body mass index (calculated as weight in kilograms divided by height in meters squared).

^a^ The standardized mean difference is the difference in means between case and control participants in units of the pooled SD. Covariates with a standardized mean difference greater than 0.2 were considered to have important imbalances.

^b^ Race or ethnic group was determined from electronic health records. Other race included American Indian or Alaska Native, Asian, Native Hawaiian, Pacific Islander, and multiracial.

^c^ Table 2 presents more details on comorbidities.

^d^ One dose of influenza vaccine after August 1, 2020.

**Table 2.** Clinical Characteristics of Adolescents

Characteristic	Participants, No. (%)^a^			Standardized difference^b^
	Total (N = 542)	Case (n = 186)	Control (n = 356)	
Medical setting				
Inpatient	9 (1.7)	7 (3.8)	2 (0.6)	0.22
Outpatient	361 (66.6)	122 (65.6)	239 (67.1)	−0.03
Testing site	172 (31.7)	57 (30.6)	115 (32.3)	−0.04
Reason for testing				
SARS-CoV-2 exposure	428 (79.0)	136 (73.1)	292 (82.0)	−0.21
COVID-19–like symptoms	147 (27.1)	114 (61.3)	33 (9.3)	1.29
Other reasons^c^	60 (11.1)	15 (8.1)	45 (12.6)	−0.15
Comorbidities				
Respiratory	82 (15.1)	29 (15.6)	53 (14.9)	0.02
Neurodevelopmental	44 (8.1)	13 (7.0)	31 (8.7)	−0.06
Endocrine	25 (4.6)	8 (4.3)	17 (4.8)	−0.02
Cardiovascular	19 (3.5)	7 (3.8)	12 (3.4)	0.02
Other comorbidities^d^	23 (4.2)	6 (3.2)	17 (4.8)	−0.08
Clinical symptoms				
Cough	50 (34.0)	42 (36.8)	8 (24.2)	0.27
Fever	40 (27.2)	37 (32.5)	3 (9.1)	0.60
Congestion	49 (33.3)	44 (38.6)	5 (15.2)	0.54
Conjunctivitis	8 (5.4)	4 (3.5)	4 (12.1)	−0.32
Pharyngitis	35 (23.8)	23 (20.2)	12 (36.4)	−0.36
Loss of taste or smell	11 (7.5)	8 (7.0)	3 (9.1)	−0.08
Chest pain or dyspnea	19 (12.9)	19 (16.7)	0	0.63
Gastrointestinal symptoms	14 (9.5)	10 (8.8)	4 (12.1)	−0.11
Constitutional symptoms^e^	59 (40.1)	52 (45.6)	7 (21.2)	0.53

^a^ Percentages may not total 100 because of rounding or because categories are not exclusive.

^b^ The standardized mean difference is the difference in means between case and control participants in units of the pooled SD. Covariates with a standardized mean difference greater than 0.2 were considered to have important imbalances.

^c^ Other reasons for testing include screening prior to a medical procedure or placement (n = 39), screening for travel (n = 5), or individual requests or asymptomatic surveillance (n = 16).

^d^ Other comorbidities include gastrointestinal, kidney, or hematologic.

^e^ Constitutional symptoms include nonspecific symptoms such as fatigue, myalgias, chills, headaches, and lethargy.

Overall, 134 adolescents (25%) were fully immunized before focal time (10 case participants [5%]; 124 control participants [35%]). The median time from the first to second dose was 21 days for both case and control participants (range: case participants, 20-53 days; control participants, 20-36 days; IQR, case participants, 21-21 days; control participants, 21-22 days). Among those fully immunized, the median time between receipt of the second dose and focal time was 62 days (range, 17 to 129 days). Nine adolescents (2%) were hospitalized (7 case participants [4%]; 2 control participants [1%]) (Table 2). One patient with COVID-19 was admitted to an intensive care unit. None of the 10 cases with breakthrough infections were hospitalized (eTable 2 in Supplement 1).

### Estimates of the VE

**Figure 2.**
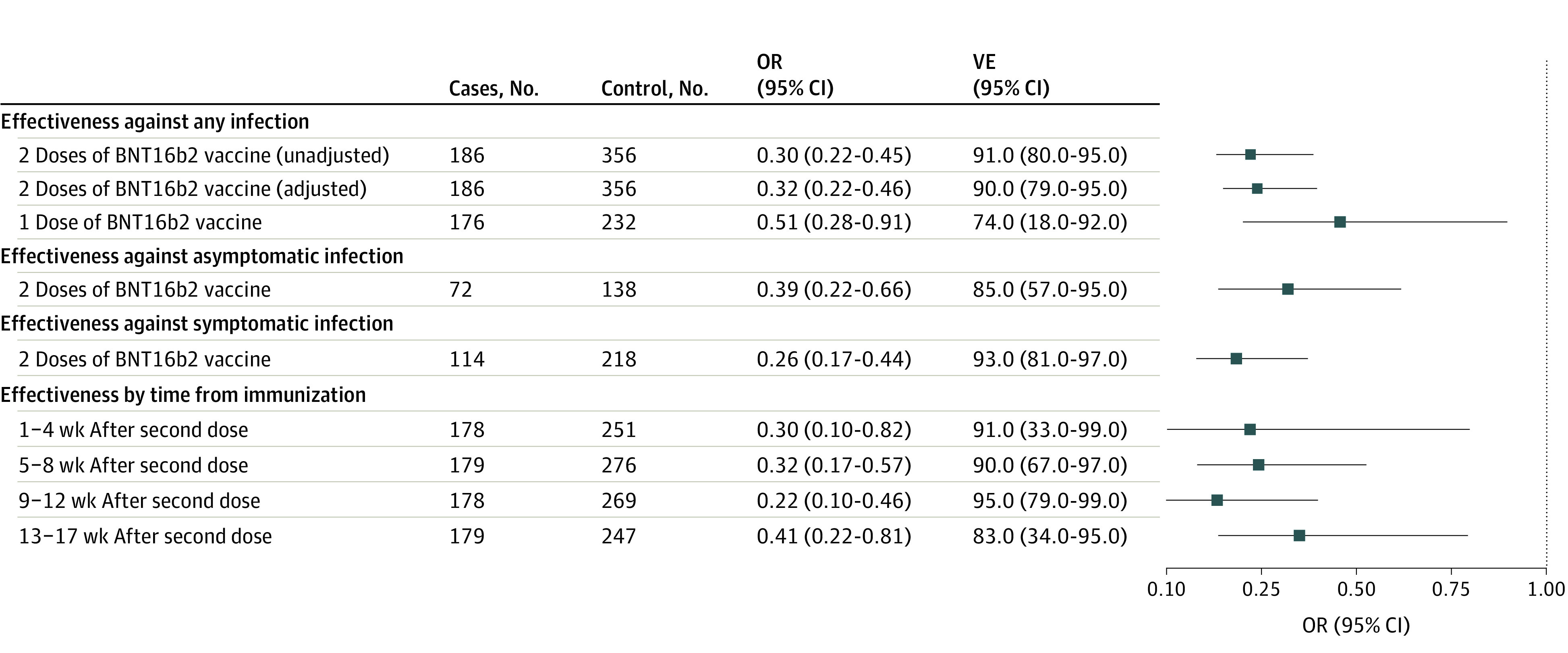
Estimated Vaccine Effectiveness (VE) Against SARS-CoV-2 Infection Shown is the overall estimated VE of 2 doses and 1 dose of the BNT162b2 vaccine and VE stratified by symptomatic status and time from immunization. Adjusted models control for race and ethnicity, insurance, and exposure to SARS-CoV-2. Whiskers indicate 95% CIs. OR indicates odds ratio.

In the unadjusted model, the VE against any infection with SARS-CoV-2 for fully immunized adolescents was 91% (95% CI, 80%-96%); for partly immunized adolescents, 74% (95% CI, 18%-92%). After the second dose, estimated VE against any infection peaked between 9 and 12 weeks (94%; 95% CI, 79%-99%) and was its lowest between 13 and 17 weeks (83%; 95% CI, 34%-96%) (Figure 2). The aVE after 2 doses was 90% (95% CI, 69%-94%). Two doses of the vaccine were slightly less effective against asymptomatic infection (VE, 85%; 95% CI, 57%-95%). Results of sensitivity analyses using models derived from different approaches were similar to those of the primary analysis (eFigure 3 and eMethods in Supplement 1). In an analysis restricted to cases infected with the Delta variant, the VE after 2 doses was 94% (95% CI, 75%-98%). The proportion of case and control participants who had received an influenza vaccine during the preceding respiratory season were the same (45 [24%] and 87 [24%]).

## Discussion

BNT162b2 was the only COVID-19 vaccine authorized for use in children younger than 18 years during the study period.^8^ Although BNT162b2 was found to be efficacious in adolescents in a clinical trial,^9^ questions remain about its effectiveness in this age group in the general population. In this matched case-control study conducted in a diverse sample of Connecticut residents, we found that the BNT162b2 vaccine was effective at preventing SARS-CoV-2 infections in adolescents aged 12 to 18 years.

Our estimates of effectiveness are consistent with those of the prelicensure trial^9^ and with a recently reported observational cohort study of adolescents in Israel, where investigators found the effectiveness of the BNT162b2 vaccine against SARS-CoV-2 infection was 66% (95% CI, 59%-72%) after the first dose and 90% (95% CI, 88%-92%) after the second dose.^33^ However, the duration of follow-up in that study was brief (<4 weeks). We found that the point estimate of VE was lowest (83%; 95% CI, 34%-96%) 13 to 17 weeks after the second dose (the latest period after focal time that we were able to evaluate). Given the overlapping confidence intervals, these VE estimates should be interpreted with caution. However, they are consistent with reports in adults, which indicated waning of VE months after completing the vaccine series.^12,13,16^ These data highlight the importance of monitoring the VE over time.

Several studies in adults have demonstrated that SARS-CoV-2 vaccines also effectively prevent transmission.^34,35^ We found the estimated VE in adolescents to be high against both symptomatic and asymptomatic infections (93% vs 85%, respectively). Prevention of asymptomatic infection is particularly important in adolescents because they are more likely than adults to be asymptomatic when infected and may be more likely to unknowingly spread the infection to others.^36,37^

### Limitations

This study has limitations. The potential for residual confounding is a limitation in all observational studies. However, our study design and analytic approach minimized the risk of such confounding. For example, ensuring control participants had a negative test result reduces bias due to misclassification of outcomes and to health care–seeking behavior.^38,39^ We also incorporated individual matching by the date of testing, age, and county of residence. This allows for better comparability for the time an adolescent was eligible to receive the vaccine and reduces potential bias from community-level differences in the prevalence of SARS-CoV-2 and in access to the vaccine.^40^ Moreover, the proportions of case participants and of matched control participants who had received the influenza vaccine in the preceding respiratory season were nearly identical, which suggests that residual confounding related to access or use of care was not a major problem. Misclassification of immunization status may have occurred if doses of the vaccine were administered at a site unaffiliated with YNHHS or outside of Connecticut. However, if misclassification occurred, its effect would be negligible and not differential, as we independently reviewed immunization records and considered the same number of sources for both cases and controls. Sequencing of the virus from all cases was not possible. However, 89% of the specimens that were sequenced were the Delta variant, which is consistent with results of population-based sequencing of the virus in Connecticut during the study period. Because there were relatively few adolescents who were partly immunized, the estimates of effectiveness after 1 dose should be interpreted with caution. Furthermore, we were not able to stratify our estimates of VE by racial and ethnic subgroups. Given that the burden of SARS-CoV-2 has been disproportionately high among underserved communities,^41,42^ more research is needed to ensure the vaccines are having an equitable impact.

## Conclusions

In this retrospective matched case-control study of US adolescents, we found that the BNT162b2 vaccine was effective in preventing both symptomatic and asymptomatic infection with SARS-CoV-2 in adolescents. The estimated effectiveness was high even against cases with the Delta variant.

## Notes

Supplement 1. **eTable 1.** Study Definitions for Study on the Effectiveness of SARS-CoV-2 Vaccines **eTable 2.** Characteristics of Cases by Vaccination Status **eFigure 1.** Weekly Number of Adolescents With Positive SARS-CoV-2 Tests and Estimated Frequency of Delta (B.1.617.2) Variants in Connecticut **eFigure 2.** Geographic Distribution of Adolescents With Positive SARS-CoV-2 Tests by County in Connecticut **eFigure 3.** Comparison Between Variable Selection Approaches **eMethods.** Supplemental Methods
eReferences.

Supplement 2.
Members of the Yale SARS-CoV-2 Genomic Surveillance Initiative
