# Supplementary material for: Assessment of Clinical Effectiveness of BNT162b2 COVID-19 Vaccine in US Adolescents
Source: JAMA Netw Open. 2022 Mar 3;5(3):e220935. doi: 10.1001/jamanetworkopen.2022.0935 (PMC8895259; doi:10.1001/jamanetworkopen.2022.0935)
Supplement: Supplement 1. — eTable 1. Study Definitions for Study on the Effectiveness of SARS-CoV-2 Vaccines eTable 2. Characteristics of Cases by Vaccination Status eFigure 1. Weekly Number of Adolescents With Positive SARS-CoV-2 Tests and Estimated Frequency of Delta (B.1.617.2) Variants in Connecticut eFigure 2. Geographic Distribution of Adolescents With Positive SARS-CoV-2 Tests by County in Connecticut eFigure 3. Comparison Between Variable Selection Approaches eMethods. Supplemental Methods eReferences. [file jamanetwopen-e220935-s001.pdf]

Supplemental Online Content

Oliveira CR, Niccolai LM, Sheikha H, et al; Yale SARS-CoV-2 Genomic Surveillance Initiative. Assessment of clinical effectiveness of BNT162b2 COVID-19 vaccine in US adolescents. *JAMA Netw Open.* 2022;5(3):e220935. doi:10.1001/jamanetworkopen.2022.0935

eTable 1. Study Definitions for Study on the Effectiveness of SARS-CoV-2 Vaccines

eTable 2. Characteristics of Cases by Vaccination Status

eFigure 1. Weekly Number of Adolescents With Positive SARS-CoV-2 Tests and Estimated Frequency of Delta (B.1.617.2) Variants in Connecticut

eFigure 2. Geographic Distribution of Adolescents With Positive SARS-CoV-2 Tests by County in Connecticut

eFigure 3. Comparison Between Variable Selection Approaches

eMethods. Supplemental Methods

eReferences.

This supplemental material has been provided by the authors to give readers additional information about their work.

© 2022 Oliveira CR et al. *JAMA Network Open.*

eTable 1. Study Definitions for Study on the Effectiveness of SARS-CoV-2 Vaccines

**Covid-19 vaccine**	• BNT162b2 (Pfizer–BioNTech) mRNA vaccine
**Study Population**	• Residents of Connecticut
	• 12-18 years of age
	• Had a medical encounter in the Yale New Haven Health System between 6/1/21 and 8/15/21.
	• Had a nasopharyngeal SARS-CoV-2 test by polymerase chain reaction (PCR) performed between 6/1/21 and 8/15/21
**Case**	• Had positive nasopharyngeal SARS-CoV-2 test by PCR
**Control**	• Individuals with a negative SARS-CoV-2 test by PCR matched to a case by age (±1 year), focal time (±1 week), and geographic area (same county).
**Focal time**	• The common date between cases and controls. The focal time was set as the date of onset of symptoms if symptomatic or the date of their SARS-CoV-2 test if asymptomatic.
**Exclusion criteria**	• Significant immunosuppression (because of either an illness or a medication)
	• Prior Covid-19 diagnosis or prior positive SARS-CoV-2 test
	• Patients who explicitly opted out of research
**Fully immunized**	• Documented receipt of $\geq$2 doses of SARS-Cov-2 vaccine at least 14 days before focal time.
**Partly immunized**	• Documented receipt of 1 dose of SARS-Cov-2 vaccine at least 14 days before focal time and no record of a second dose or the second dose occurred <14 days before focal time.
**Covid-19-like illness**	• Having one or more of the following: measured or subjective fever, cough, dyspnea, headache, fatigue, myalgia, sore throat, coryza, nausea/vomiting, diarrhea, altered mental status, loss of taste or loss of smell.
**Significant immunosuppression**	• Having one or more of the following: Newly diagnosed cancer in the past six months, HIV infection, congenital immunodeficiency, asplenia, previous solid organ or bone marrow transplant.<br>• Having received treatment with one of the following immunosuppressive medications within the past six months: Tacrolimus, Mycophenolate Motefil, Rituximab, Azathioprine, Basiliximab, Cyclophosphamide, Cyclosporine, Azathioprine, Adalimumab, Anakinra, Etanercept, Infliximab, Methotrexate, Sirolimus, or Corticosteroids.
**SARS-CoV-2 exposure**	• Exposure to a case of SARS-CoV-2 in the last 14 days (e.g., household, school, crowding, travel histories). Exposure had to be documented in the medical record

© 2022 Oliveira CR et al. *JAMA Network Open.*

eTable 2. Characteristics of Cases by Vaccination Status, N=186

Characteristic	Total (N=186)	BNT162B2 Covid-19 Vaccine		SMD[a]
		No doses (N=173)	At least 1 dose (N=13)	
**Age, median (IQR), yr**	14 (13-16)	14 (13-16)	16 (14-16)	0.99
**Sex**				
Female	86 (46.2%)	77 (44.5%)	9 (69.2%)	0.74
Male	100 (53.8%)	96 (55.5%)	4 (30.8%)	-0.74
**Race or ethnic group**				
Black, non-Hispanic	34 (18.3%)	34 (19.7%)	0 (0.0%)	-0.64
Hispanic or Latinx	37 (19.9%)	34 (19.7%)	3 (23.1%)	-0.82
White, non-Hispanic	92 (49.5%)	83 (48.0%)	9 (69.2%)	1.02
Other race[b]	14 (7.5%)	13 (7.5%)	1 (7.7%)	0.16
Unknown	9 (4.8%)	9 (5.2%)	0 (0.0%)	-0.37
**Health Insurance**				
Private	88 (47.3%)	77 (44.5%)	11 (84.6%)	1.00
Government	67 (36.0%)	65 (37.6%)	2 (15.4%)	-0.61
Uninsured or unknown	31 (16.7%)	31 (17.9%)	0 (0.0%)	-0.64
**Month sample was collected**				
June	24 (12.9%)	21 (12.1%)	3 (23.1%)	0.40
July	64 (34.4%)	61 (35.3%)	3 (23.1%)	-0.59
August	98 (52.7%)	91 (52.6%)	7 (53.8%)	0.19
**Medical setting**				
Inpatient	7 (3.8%)	7 (4.0%)	0 (0.0%)	-0.24
Outpatient	122 (65.6%)	114 (65.9%)	8 (61.5%)	-0.17
Testing Site	57 (30.6%)	52 (30.1%)	5 (38.5%)	0.23
**Comorbidities**				
Any comorbidities	63 (33.9%)	61 (35.3%)	2 (15.4%)	-1.03
BMI > 95 percentile[c]	30 (16.1%)	30 (17.3%)	0 (0.0%)	-0.66
Respiratory	29 (15.6%)	29 (16.8%)	0 (0.0%)	-0.57
Neurodevelopmental	13 (7.0%)	11 (6.4%)	2 (15.4%)	-0.31
Endocrine	8 (4.3%)	8 (4.6%)	0 (0.0%)	-0.28
Cardiovascular	7 (3.8%)	7 (4.0%)	0 (0.0%)	-0.31
Other[d]	6 (3.2%)	6 (3.5%)	0 (0.0%)	-0.34

© 2022 Oliveira CR et al. *JAMA Network Open.*

Characteristic	Total (N=186)	BNT162B2 Covid-19 Vaccine		SMD[a]
		No doses (N=173)	At least 1 dose (N=13)	
**Clinical symptoms -- no. (%)**				
Any symptom	114 (61.3%)	106 (61.3%)	8 (61.5%)	0.01
Cough	42 (36.8%)	41 (38.7%)	1 (12.5%)	-0.61
Fever	37 (32.5%)	35 (33.0%)	2 (25.0%)	-0.17
Congestion	44 (38.6%)	40 (37.7%)	4 (50.0%)	0.24
Conjunctivitis	4 (3.5%)	4 (3.8%)	0 (0.0%)	-0.28
Pharyngitis	23 (20.2%)	23 (21.7%)	0 (0.0%)	-0.74
Loss of taste or smell	8 (7.0%)	8 (7.5%)	0 (0.0%)	-0.40
Chest pain or dyspnea	19 (16.7%)	19 (17.9%)	0 (0.0%)	-0.66
Gastrointestinal symptoms	10 (8.8%)	9 (8.5%)	1 (12.5%)	0.13
Constitutional symptoms[e]	52 (45.6%)	49 (46.2%)	3 (37.5%)	-0.17
**Exposed to SARS-CoV-2**	136 (73.1%)	125 (72.3%)	11 (84.6%)	0.13
**Healthcare utilization**				
At least 1 prior influenza vaccine dose[f]	30 (16.1%)	27 (15.6%)	3 (23.1%)	0.12
Medical visits after 1/1/20 -- median (range)	2.0 (0.0-4.0)	1.0 (0.0-4.0)	2.0 (2.0-6.0)	0.38

Percentages may not total 100 because of rounding.

[a] SMD denotes the standardized mean difference. It is the difference in means between cases and controls in units of the pooled standard deviation. Covariates with SMD >0.2 were considered to have important imbalances.

[b] Race or ethnic group was determined from electronic health records. Other race included Asian, American Indian or Alaska native, native Hawaiian, pacific islander, and mixed race.

[c] Other comorbidities include gastrointestinal, renal, or hematologic.

[d] The BMI denotes body-mass index is the weight in kilograms divided by the square of the height in meters

[e] One dose of influenza vaccine after August 1, 2020

[f] Constitutional symptoms include non-specific symptoms like fatigue, myalgias, chills, headaches, and lethargy

© 2022 Oliveira CR et al. *JAMA Network Open.*

eFigure 1. Weekly Number of Adolescents With Positive SARS-CoV-2 Tests and Estimated Frequency of Delta (B.1.617.2) Variants in Connecticut

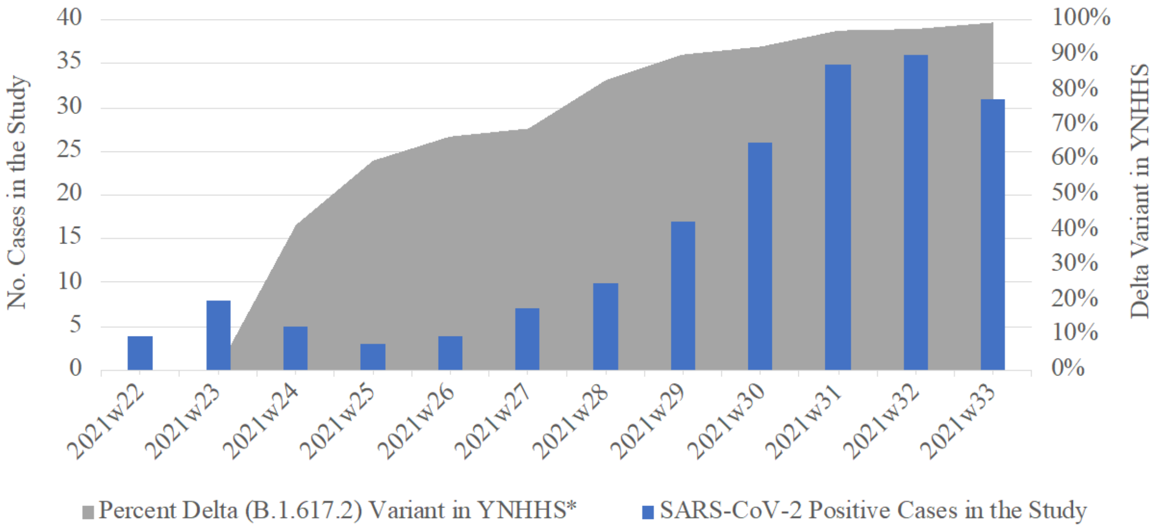

Estimated temporal frequencies of Delta (B.1.617.2) variant in YNHHS identified through unbiased sequencing of adults and children and reported by the Connecticut Department of Public Health.

© 2022 Oliveira CR et al. *JAMA Network Open.*

eFigure 2. Geographic Distribution of Adolescents With Positive SARS-CoV-2 Tests by County in Connecticut

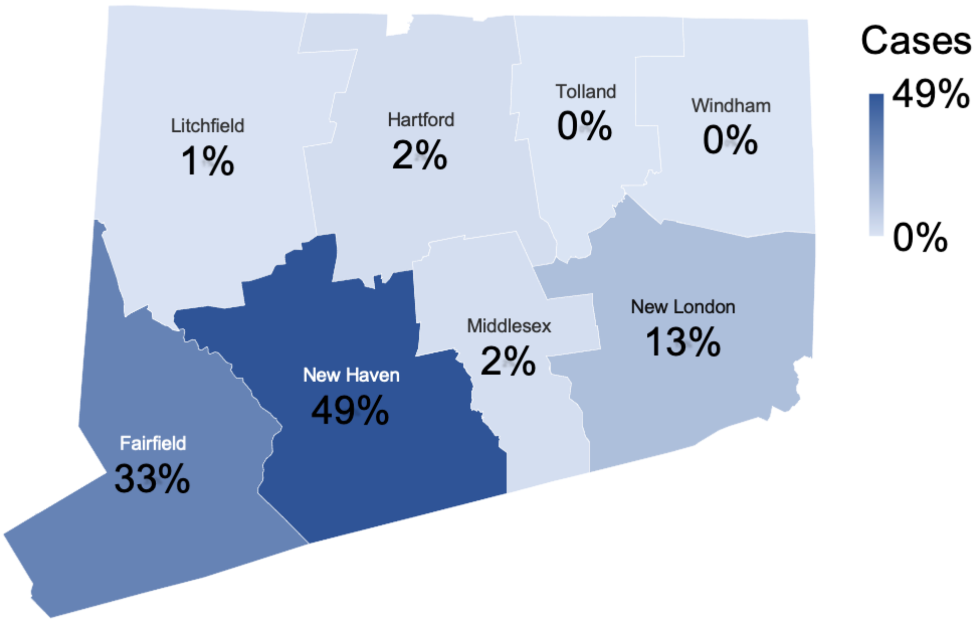

© 2022 Oliveira CR et al. *JAMA Network Open.*

eFigure 3. Comparison Between Variable Selection Approaches

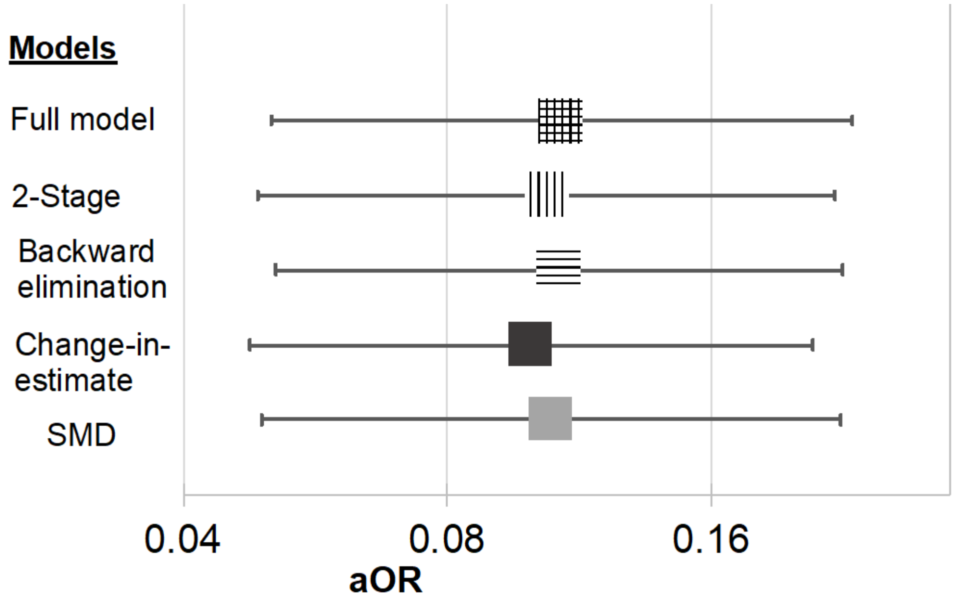

Models	Variables	Log Odds	SE	aOR
Full model	All variables	-2.22	0.392	0.11
2-Stage	Age, Race, Insurance	-2.26	0.385	0.10
Backward elimination	Age, Insurance	-2.23	0.379	0.11
Change-in-estimate	Age	-2.30	0.377	0.10
SMD	Race, Insurance, Covid-19 exposure	-2.25	0.388	0.11

Data are presented as effectiveness point estimates, with bars indicating the corresponding 95% confidence intervals. SE denotes standard error; aOR denotes adjusted odds ratio. Details of model-selection approaches can be found in Section 3 of Supplemental Methods.

© 2022 Oliveira CR et al. *JAMA Network Open.*

eMethods. Supplemental Methods

Sample size and power

Using established formulas, we calculated sample sizes for different proportions of controls that might be vaccinated and for a 1:1, 2:1, and 3:1 ratio of controls to cases.[1, 2] The number of cases needed to detect a range of estimates of the vaccine's effectiveness with $\alpha < 0.05$ and a power of 80-90% are presented below.

Though it was not known what the vaccine uptake would be in this subgroup of the population, *a priori*, we extrapolated from the national surveys that found approximately a third of adolescents <18 years of age were fully immunized as of July 2021.[3] Assuming that >30% of controls would be fully immunized, our study, with 186 case-control sets (2:1 ratio), was powered (≥ 90%) to detect a minimal effectiveness of 50% (two-tailed alpha < 0.05).

Prevalence of Vaccination in Controls	Power	Number of cases needed to detect an effectiveness of:											
		70%			60%			50%			40%		
		Controls/Case			Controls/Case			Controls/Case			Controls/Case		
		1	2	3	1	2	3	1	2	3	1	2	3
20%	80%	102	81	74	159	124	113	256	198	179	443	340	305
	90%	132	103	93	208	161	145	339	260	233	590	448	401
30%	80%	72	56	51	113	87	79	185	142	127	323	246	220
	90%	93	72	65	148	114	102	244	186	166	430	326	291
40%	80%	57	44	40	92	70	63	152	116	103	270	204	182
	90%	74	57	51	120	92	82	201	153	136	359	271	242
50%	80%	50	38	34	81	61	55	137	103	92	247	186	165
	90%	64	49	44	106	81	72	181	137	122	328	247	220

Modeling Approach

Unadjusted and adjusted odds ratios (OR), along with their 95% confidence intervals (CI), were estimated using conditional logistic regression. The vaccine's effectiveness (VE) was calculated as (1 − OR) x 100%. The log odds of SARS-CoV-2 infection for individuals in matched sets was modeled using the following conditional logistic model:

$$\mathbf{Log(odds_{case}) = \alpha_i + \beta_e(vax) + \beta_1(var\_1) + \beta_2(var\_2) + ... + \beta_k(var\_k)}$$

where αi is the stratum-specific constant term for each matched set, βe is the coefficient denoting immunization status, and $\beta 1$ through βk are the coefficients for each potential confounder included in the model. For the primary analysis, the immunization status was coded as '1' if the patient received two doses of SARS-CoV-2 vaccine 14 days before focal time and as '0' otherwise (including unvaccinated). Our modeling strategy was to create models that always contain the immunization status and additional potential confounders as needed. Missing data were reported or included within a level of a categorical variable (i.e., unknown race/ethnicity).

To assess for variation in the VE by the number of doses received, we created a categorical variable coded as '1' if the patient received only one dose, '2' if they received two doses, and '0' otherwise (including unvaccinated). Exponentiating the beta coefficient for the different levels of categorical vaccine variable yields the OR for those given one or two doses compared with unvaccinated. To test if there are significant differences between one and two doses (i.e., pair-wise comparisons), we recoded the categorical vaccine variable using two

© 2022 Oliveira CR et al. *JAMA Network Open.*

doses as the referent category and interpreted the corresponding beta coefficients, as previously described.[4, 5] To assess the effect of time since immunization on the effectiveness of the vaccine, four new terms were created to account for time in 4-week intervals (i.e., 1-4 weeks, 5-8 weeks, 9-12 weeks, and 13-17 weeks prior to focal time). These terms equaled to 1 for patients who were immunized a given number of weeks prior to focal time (e.g., 1-4 weeks) and 0 for everybody else, including all unvaccinated patients.

Comparing Model Selection Approaches

To address model-selection uncertainty, we compared the adjusted VE (aVE) and the vaccine coefficient's error terms (on the logit scale) using different model-selection approaches: the full model, the two-stage selection, stepwise backward elimination (using p-value), change-in-estimate method, and standardized mean difference method (SMD). The full model adjusted for nine potential confounders: age, sex, race/ethnicity, health insurance, at least one comorbidity, obesity, the month of the year sample was obtained, the number of medical visits after 1/1/20, and recent exposure to Covid-19. After fitting the full model, we first tested the two-stage approach. In the two-stage method, each of the potential confounders was considered separately using conditional logistic regression models, and a multivariable model was built by including all statistically significant variables using a p-value <0.05 as the cutoff.[6] Three variables (age, race/ethnicity, and insurance status) were selected using this approach. The results of the two-stage selection approach are shown below.

The backward elimination began with the "full model" (all potential confounders), and then each potential confounder was eliminated, one at a time (least significant first), based on whether it had a p-value cutoff of <0.05 [7]. Two variables were selected (age and insurance status) using the backward elimination approach. For the change-in-estimate approach, potential confounders were selected in a stepwise fashion, and variables were eliminated using a backward strategy if its inclusion caused a change in the VE measurement of <5%.[8] Using the change-in-estimate approach, only the variable for age was maintained in the multivariate model. For the SMD approach, multivariable models were built similar to the 2-stage method; however, an SMD >0.2 was used as the cutoff (rather than p-value).[9] Using this approach led to the inclusion of variables for race/ethnicity, insurance status, and SARS-CoV-2 exposure. The adjusted OR and standard errors were similar regardless of the model selection approach that was used (eFigure 3).

Two-stage variable selection

Models: Stage 1	OR	95% CI	*P value*
**Model 1**			
Fully immunized	0.1	[0.05,0.21]	<0.01
Age, yr	0.7	[0.53,0.93]	0.014
**Model 2**			
Fully immunized	0.09	[0.05,0.20]	<0.01
Female sex	1.01	[0.68,1.48]	0.97
**Model 3**			
Fully immunized	0.1	[0.05,0.20]	<0.01
Race or ethnic group			
Black, non-Hispanic	1.12	[0.65,1.93]	0.68
Hispanic or Latinx	1.47	[0.84,2.58]	0.17
White, non-Hispanic		reference group	
Other race[a]	0.5	[0.26,0.98]	0.042
**Model 4**			
Fully immunized	0.1	[0.05,0.21]	<0.01
Health Insurance			
Private insurance		reference group	
Government insurance	1.46	[0.94,2.28]	0.090
Uninsured or unknown	1.82	[1.05,3.18]	0.034
**Model 5**			
Fully immunized	0.1	[0.05,0.20]	<0.01
Any comorbidities[b]	0.92	[0.60,1.40]	0.69
**Model 6**			
Fully immunized	0.09	[0.04,0.20]	<0.01
BMI > 95 percentile[c]	1.26	[0.72,2.19]	0.42
**Model 7**			
Fully immunized	0.1	[0.05,0.20]	<0.01
Month sample was collected			
June		reference group	
July	1.6	[0.40,6.42]	0.50
August	2.38	[0.49,11.55]	0.28

© 2022 Oliveira CR et al. *JAMA Network Open.*

Model 8	OR	95% CI	*P value*
Fully immunized	0.1	[0.05,0.20]	<0.01
Exposed to SARS-CoV-2	0.67	[0.41,1.11]	0.11
**Model 9**			
Fully immunized	0.1	[0.05,0.20]	<0.01
Medical visits after 1/1/20	0.99	[0.96,1.02]	0.60
**Final Model: Stage 2**			
Fully immunized	0.1	[0.05,0.22]	<0.01
Age, yr	0.68	[0.51,0.92]	0.013
Race or ethnic group			
Black, non-Hispanic	0.97	[0.54,1.74]	0.91
Hispanic or Latinx	1.32	[0.74,2.35]	0.34
White, non-Hispanic		reference group	
Other race[a]	0.48	[0.24,0.96]	0.036
Health Insurance			
Private insurance	reference group		
Government insurance	1.35	[0.82,2.23]	0.23
Uninsured or unknown	1.87	[1.06,3.28]	0.029

*P value*s for odds ratio (OR) estimated using conditional logistic regression. All estimates include the variable denoting immunization status.

[a] Race or ethnic group was determined from electronic health records. Other race included Asian, American Indian or Alaska native, native Hawaiian, pacific islander, and mixed race.

[b] Any comorbidities, see Table 2 for more details

[c] The BMI denotes body-mass index is the weight in kilograms divided by the square of the height in meters

© 2022 Oliveira CR et al. *JAMA Network Open.*

Change in estimate variable selection

Variables Removed	OR	95% CI	Change, %
**Full adjusted model**	0.11	[0.05,0.23]	-
**Removed female sex**	0.11	[0.05,0.23]	0.12%
**Removed month sample was collected**			
June	reference group		
July	0.11	[0.05,0.23]	-0.22%
August	0.11	[0.05,0.23]	-0.07%
**Removed race or ethnic group**			
Black, non-Hispanic	0.11	[0.05,0.23]	0.31%
Hispanic or Latinx	0.11	[0.05,0.23]	-0.29%
White, non-Hispanic	reference group		
Other race[a]	0.11	[0.05,0.23]	2.96%
**Removed comorbidities[b]**	0.11	[0.05,0.23]	-0.25%
**Removed BMI > 95 percentile[c]**	0.11	[0.05,0.23]	1.43%
**Removed exposed to SARS-CoV-2**	0.11	[0.05,0.23]	-1.78%
**Removed medical visits after 1/1/20**	0.11	[0.05,0.23]	-2.71%
**Removed health insurance**			
Private insurance	reference group		
Government insurance	0.10	[0.05,0.22]	-3.62%
Uninsured or unknown	0.10	[0.05,0.21]	-3.54%
**Removed age, yr**	0.09	[0.05,0.20]	-5.10%

*P value*s for odds ratio (OR) estimated using conditional logistic regression. All estimates include the variable denoting immunization status.

[a] Race or ethnic group was determined from electronic health records. Other race included Asian, American Indian or Alaska native, native Hawaiian, pacific islander, and mixed race.

[b] Any comorbidities, see Table 2 for more details

[c] The BMI denotes body-mass index is the weight in kilograms divided by the square of the height in meters

© 2022 Oliveira CR et al. *JAMA Network Open.*

Logic Behind the Use of Influenza Vaccine as a Sham Exposure

Even after matching and adjusting for potential confounders, residual selection bias can be seen if unmeasured or unidentified factors are associated with the subject's propensity to be immunized or propensity to seek medical care and the selection mechanism in the study. To assess for the potential of residual selection bias, we incorporated measurements of a "sham" exposure (i.e., a vaccine that does not affect the outcome of interest but has a similar likelihood of being received at about the same time as the vaccine of interest) into the design of this study and compared the proportion of cases and the proportion of matched controls who received the sham exposure before *focal time.*[10] Since influenza vaccines are recommended to be given to all adolescents but have no effect on the risk of developing SARS-CoV-2 infection, we expected that in the absence of residual selection bias, there would be no significant difference in the proportions who had received the vaccine.

The logic behind using a sham exposure is shown in the casual diagram below. From this diagram, we can see that if the apparent effectiveness of the vaccine is due to selection bias from an unmeasured factor related to immunization, then the sham exposure may also erroneously appear to be effective at preventing disease (i.e., SARS-CoV-2 infection in this study). On the other hand, if the proportions of cases and of matched controls that had received $\geq$1 dose of the sham vaccine (i.e., influenza vaccine in this study) were similar but there is still a significant difference in the proportions that received Covid-19 vaccine, this would suggest that the estimated effectiveness of the Covid-19 vaccine is unlikely to have been substantially affected by some uncontrolled selection bias.

Illustration of the A) Sham Exposure and, B) Effect of Conditioning on this Exposure

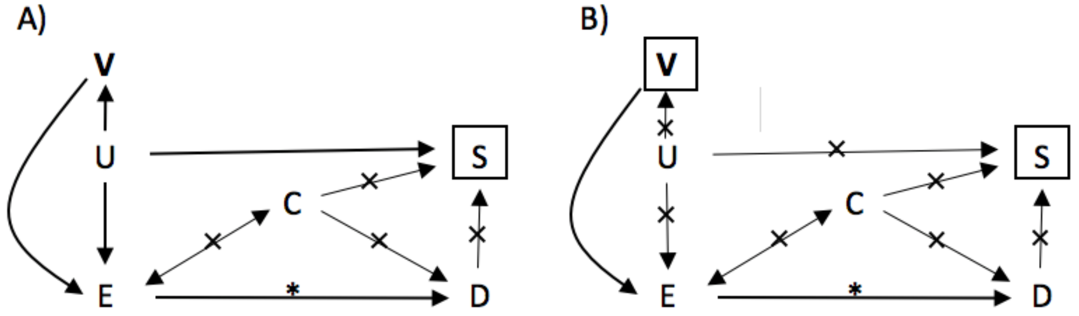

In this causal diagram, we denote the exposure of interest (Covid-19 vaccine) by the letter "E" and the outcome of interest by the letter "D." Confounding factors are represented by the letter "C," and the letter "S" represents whether a subject is selected to participate in the study. Arrows denote causal pathways between two variables. Conditioning by a particular variable is denoted by a box surrounding the letter, and an "X "over the arrow denotes a disrupted association post-conditioning. The main effect being measured in the matched case-control study (i.e., the vaccine's effectiveness) is emphasized with an Asterix above an arrow. Variable "V" is an exposure that is associated with both immunization status and the unmeasured confounder but is not associated with disease (in effect, a "sham exposure").

Sample Collection and Genomic Sequencing

Clinical samples

Samples collected were nasopharyngeal swabs or nasal swabs in viral transport media. Samples were processed and tested for SARS-Cov-2 as part of routine clinical care at YNHHS-affiliated laboratories that are certified under the Clinical Laboratory Improvement Amendments of 1988 (CLIA). For the identification of SARS-CoV-2 RNA, samples were tested using Food and Drug Administration (FDA) authorized assays, which included Xpert Xpress

SARS-CoV-2 (Cepheid, Sunnyvale, CA), Aptima SARS-CoV-2 (Hologic) BioGx SARS-CoV-2 Reagents for BD Max (Becton Dickinson, Franklin Lakes, NJ), TaqPath COVID-19 Combo Kit (ThermoFisher, Waltham, MA), and Simplexa COVID-19 Direct (Diasorin, Cypress, CA) using standard clinical procedures as previously described.[11]

Sample selection

Samples were initially selected for genomic surveillance from confirmed SARS-CoV-2 positive clinical samples obtained for routine testing provided by the Yale Clinical Virology Lab or Yale Pathology lab. We extracted RNA from 300μL of the sample using the MagMax viral/pathogen nucleic acid extraction kit (Thermo Fisher), eluting into 75μL elution buffer. We tested the extracted samples using our "variant of concern" RT-qPCR to determine the SARS-CoV-2 viral load and selected all samples with a cycle threshold <35 for sequencing.[12]

Sample preparation and sequencing

Samples were processed using a multiplexed PCR as previously described.[13] Briefly, samples were prepared in batches of 94 with negative (water) controls incorporated at extraction, cDNA synthesis, and amplicon generation. The Illumina COVIDSeq Test RUO version was used to synthesize cDNA, generate amplicons, and tagment amplicons. Amplicons were pooled and cleaned before quantification with Qubit High-Sensitivity dsDNA kit. The resulting libraries were sequenced using a 2x150 approach on an Illumina NovaSeq at the Yale Center for Genome Analysis, with each sample receiving at least 1 million reads.

Data processing

Consensus genomes were generated by aligning reads to Wuhan-Hu-1 reference genomes (GenBank MN908937.3) using BWA-MEM v0.7.15. iVar v1.2.1,[14] and SAMTools,[15] were used to trim sequencing adaptors, primer sites, and call bases at >60% frequency at each site with ≥20 reads. An ambiguous "N" was called for sites with fewer than ten reads. Negative controls were confirmed to consist of ≥95% N's in all cases. To assign lineages, samples were analyzed with Pangolin v2.4.2.[16]

eReferences.

1. Fleiss JL, Levin B, M CP. *Statistical methods for rates and proportions. 3rd ed.* John Wiley & Sons, Inc; 2003.

2. Dupont WD. Power calculations for matched case-control studies. *Biometrics*. Dec 1988;44(4):1157-68.

3. Murthy BP, Zell E, Saelee R, et al. COVID-19 Vaccination Coverage Among Adolescents Aged 12-17 Years - United States, December 14, 2020-July 31, 2021. *MMWR Morb Mortal Wkly Rep*. Sep 3 2021;70(35):1206-1213. doi:10.15585/mmwr.mm7035e1

4. Oliveira CR, Ortiz AM, Sheth SS, Shapiro ED, Niccolai LM. Effectiveness of HPV vaccine by age at vaccination and number of doses: protocol for a population-based matched case-control study. *BMJ Open*. Apr 19 2021;11(4):e043093. doi:10.1136/bmjopen-2020-043093

5. Niccolai LM, Ogden LG, Muehlenbein CE, Dziura JD, Vazquez M, Shapiro ED. Methodological issues in design and analysis of a matched case-control study of a vaccine's effectiveness. *J Clin Epidemiol*. Nov 2007;60(11):1127-31. doi:10.1016/j.jclinepi.2007.02.009

6. Greenland S. Modeling and variable selection in epidemiologic analysis. *Am J Public Health*. Mar 1989;79(3):340-9. doi:10.2105/ajph.79.3.340

7. Williams R. Step we gaily, on we go. *The Stata Journal*. 2007;7(2):272-274.

8. Weng HY, Hsueh YH, Messam LL, Hertz-Picciotto I. Methods of covariate selection: directed acyclic graphs and the change-in-estimate procedure. *Am J Epidemiol*. May 15 2009;169(10):1182-90. doi:10.1093/aje/kwp035

9. Belitser SV, Martens EP, Pestman WR, Groenwold RH, de Boer A, Klungel OH. Measuring balance and model selection in propensity score methods. *Pharmacoepidemiol Drug Saf*. Nov 2011;20(11):1115-29. doi:10.1002/pds.2188

10. Shapiro ED. Case-control studies of the effectiveness of vaccines: validity and assessment of potential bias. *The Pediatric infectious disease journal*. Feb 2004;23(2):127-31. doi:10.1097/01.inf.0000109248.32907.1d

11. Peaper DR, Murdzek C, Oliveira CR, Murray TS. Severe Acute Respiratory Syndrome Coronavirus 2 Testing in Children in a Large Regional US Health System During the Coronavirus Disease 2019 Pandemic. *The Pediatric infectious disease journal*. Mar 1 2021;40(3):175-181. doi:10.1097/INF.0000000000003024

12. Vogels CBF, Breban MI, Ott IM, et al. Multiplex qPCR discriminates variants of concern to enhance global surveillance of SARS-CoV-2. *PLoS Biol*. May 2021;19(5):e3001236. doi:10.1371/journal.pbio.3001236

13. Vogels CBF, Brito AF, Wyllie AL, et al. Analytical sensitivity and efficiency comparisons of SARS-CoV-2 RT-qPCR primer-probe sets. *Nat Microbiol*. Oct 2020;5(10):1299-1305. doi:10.1038/s41564-020-0761-6

14. Grubaugh ND, Gangavarapu K, Quick J, et al. An amplicon-based sequencing framework for accurately measuring intrahost virus diversity using PrimalSeq and iVar. *Genome Biol*. Jan 8 2019;20(1):8. doi:10.1186/s13059-018-1618-7

15. Danecek P, Bonfield JK, Liddle J, et al. Twelve years of SAMtools and BCFtools. *Gigascience*. Feb 16 2021;10(2)doi:10.1093/gigascience/giab008

16. O'Toole A, Scher E, Underwood A, et al. Assignment of epidemiological lineages in an emerging pandemic using the pangolin tool. *Virus Evol*. 2021;7(2):veab064. doi:10.1093/ve/veab064

© 2022 Oliveira CR et al. *JAMA Network Open.*
