# Supplementary material for: Assessment of Clinical Effectiveness of BNT162b2 COVID-19 Vaccine in US Adolescents
Source: JAMA Netw Open. 2022 Mar 3;5(3):e220935. doi: 10.1001/jamanetworkopen.2022.0935 (PMC8895259; doi:10.1001/jamanetworkopen.2022.0935)
Supplement: Supplement 2. — Members of the Yale SARS-CoV-2 Genomic Surveillance Initiative [file jamanetwopen-e220935-s002.pdf]

Supplemental Online Content: Nonauthor Collaborators

*Indicates required information. Only first name, last name, and suffix will appear in PubMed.

*Group Name(s): Yale SARS-CoV-2 Genomic Surveillance Initiative							
***First Name and Middle Initial(s)**	***Last Name**	***Suffix (eg, Jr, III)**	Academic Degrees	Institution	Location (city, state/province, country)	Role or Contribution, eg, chair, principal investigator	Group (if more than 1 Group listed in the byline) and/or Subgroup (eg, Steering Committee)
Kendall	Billig			Department of Epidemiology of Microbial Diseases, Yale School of Public Health.	New Haven, CT 06510, USA		
Mallery I.	Breban			Department of Epidemiology of Microbial Diseases, Yale School of Public Health.	New Haven, CT 06510, USA		
Anderson	Brito		PhD	Department of Epidemiology of Microbial Diseases, Yale School of Public Health.	New Haven, CT 06510, USA		
Rebecca	Earnest		MPH	Department of Epidemiology of Microbial Diseases, Yale School of Public Health.	New Haven, CT 06510, USA		
Joseph R.	Fauver		PhD	Department of Epidemiology of Microbial Diseases, Yale School of Public Health.	New Haven, CT 06510, USA		
Tobias	Koch			Department of Epidemiology of Microbial Diseases, Yale School of Public Health.	New Haven, CT 06510, USA		
Isabel	Ott			Department of Epidemiology of Microbial Diseases, Yale School of Public Health.	New Haven, CT 06510, USA		
Mary	Petrone			Department of Epidemiology of Microbial Diseases, Yale School of Public Health.	New Haven, CT 06510, USA		
Chantal B.F.	Vogels		PhD	Department of Epidemiology of Microbial Diseases, Yale School of Public Health.	New Haven, CT 06510, USA		
Kien	Pham		PhD	Department of Epidemiology of Microbial Diseases, Yale School of Public Health.	New Haven, CT 06510, USA		
Irina	Tikhonova		MSc	Yale Center for Genome Analysis, Yale University.	New Haven, CT 06510, USA		
Christopher	Castaldi		MSc	Yale Center for Genome Analysis, Yale University.	New Haven, CT 06510, USA		
Shrikant	Mane		PhD	Yale Center for Genome Analysis, Yale University.	New Haven, CT 06510, USA		
Kaya	Bilguvar		MD	Yale Center for Genome Analysis, Yale University.	New Haven, CT 06510, USA		

*Indicates required information. Only first name, last name, and suffix will appear in PubMed.

***First Name and Middle Initial(s)**	***Last Name**	***Suffix (eg, Jr, III)**	Academic Degrees	Institution	Location (city, state/province, country)	Role or Contribution, eg, chair, principal investigator	Group (if more than 1 Group listed in the byline) and/or Subgroup (eg, Steering Committee)
Bony	De Kumar			Yale Center for Genome Analysis, Yale University.	New Haven, CT 06510, USA		
David	Ferguson		MSc	Center for Outcomes Research and Evaluation, Yale New Haven Hospital.	New Haven, CT 06510, USA		
Nicholas	Kerantzas			Department of Laboratory Medicine, Yale New Haven Hospital.	New Haven, CT 06510, USA		
Marie	Landry		MD	Departments of Laboratory Medicine and Medicine, Yale University School of Medicine.	New Haven, CT 06510, USA		
David	Peaper		MD, PhD	Departments of Laboratory Medicine and Medicine, Yale University School of Medicine.	New Haven, CT 06510, USA		
Wade	Schulz		MD, PhD	Department of Laboratory Medicine, Yale School of Medicine, New Haven, CT 06510, USA; Center for Outcomes Research and Evaluation, Yale New Haven Hospital.	New Haven, CT 06510, USA		
